# Draft Genome Sequence of Five Shiga Toxin-Producing *Escherichia coli* Strains Isolated from Wild Deer in Japan

**DOI:** 10.1128/genomeA.01455-16

**Published:** 2017-03-02

**Authors:** Hiroshi Asakura, Tetsuya Ikeda, Shiori Yamamoto, Hidenori Kabeya, Hiromu Sugiyama, Shinji Takai

**Affiliations:** a Division of Biomedical Food Research, National Institute of Health Sciences, Tokyo, Japan; b Department of Infectious Diseases, Hokkaido Prefectural Institute of Public Health, Hokkaido, Japan; c Department of Veterinary Public Health, Nihon University, Kanagawa, Japan; d Division of Parasitology, National Institute of Infectious Diseases, Tokyo, Japan; e Laboratory of Animal Hygiene, Kitasato University, Aomori, Japan

## Abstract

Shiga toxin-producing *Escherichia coli* (STEC) is one of the major foodborne pathogens. Having observed the wide distribution of this pathogen in wild deer, we report here the draft genome sequence of five STEC strains isolated from wild deer (*Cervus nippon yesoensis*) in Hokkaido, Japan.

## GENOME ANNOUNCEMENT

Shiga toxin-producing *Escherichia coli* (STEC) represents a major issue for public health because of its capability to cause large outbreaks and the severity of the associated illnesses (1). Epidemiological data have mounted evidence for the distribution of STEC in farm animals (2), but wildlife animals such as wild deer and other cervids and birds also carry STEC (3–5). Recently, increased trends for the consumption of game meats, mainly consisting of wild deer and boar meats, in Japan (6) have made it necessary to elucidate virulence properties of STEC from these wildlife animals. As wild deer has been recognized as one of the major reservoirs (7), we obtained STEC strains 11226, 11229, 11247, 15821, and 16309 from feces of wild deer (*Cervus nippon yesoensis*) inhabiting Hokkaido, Japan, between 2011 and 2016. Genomic DNA of the five strains were sequenced by single-end sequencing with an Ion Torrent PGM sequencer (Thermo Fisher Scientific, Waltham, MA, USA), resulting in an average coverage of 139×. Raw reads were trimmed and *de novo* assembled using CLC Genomics Workbench v 9.0 (Qiagen, Hilden, Germany). The parameters for trimming were as follows: ambiguous limit, 2; quality limit, 0.05; number of 5′-terminal nucleotides, 20; number of 3′-terminal nucleotides, 5. The parameters for the *de novo* assembly were as follows: mapping mode, create simple contig sequences (fast); bubble size, 50; word size, 21; minimum contig length, 1,000 bp; perform scaffolding, no; autodetect paired distances, yes.

The draft genomes of the five STEC strains were assembled into 250, 246, 282, 238, and 135 contigs with an accumulated length ranging from 5,248,909 to 5,404,906 bp (*N*_50_, 140,330 bp on average) and an average G+C content of 45.5% to 50.7%. The genome was annotated by the RAST server (8). Annotation of these assemblies identified 5,161 to 5,401 coding sequences (CDSs), 70 to 79 rRNAs, and 64 to 70 tRNAs.

Their sequence types (ST) and serotypes were also identified as follows: ST32/O145:NM (11226), ST446/OUT:HUT (11229), ST11/O157:H7 (11247), ST32/O145:NM (15821), and ST5597/OUT:HUT (16309), by multilocus sequence type (MLST) 1.8 (https://cge.cbs.dtu.dk/services/) and slide agglutination testing.

Three genomes (strains 11226, 15821, and 16309) contained *stx1a*, and two strains, 11229 and 11247, contained *stx2d*, which showed 100% similarity in the nucleotide sequences to those from STEC previously isolated from wild deer (3). Other representative virulence genes, *eae* and *ehxA*, were present in three genomes (11226, 11247, and 15821). Since *eae*-negative STEC also cause human illness (9), we could not exclude the possibility for their potential to cause human infection. Additionally, four genomes except for strain 11229 contained *astA*, which is associated with the development of diarrhea (10). The data provided can aid in future efforts to identify the source of infection. Further accumulation of genomic data of the deer-originating STEC and their use for evolutional studies would also improve our understandings of the host or geographic adaptation of this pathogen.

### Accession number(s).

This whole-genome shotgun project has been deposited at DDBJ/EMBL/GenBank under the GenBank accession numbers BDLI01000000 (11226), BDLJ01000000 (11229), BDLK01000000 (11247), BDLL01000000 (15821), and BDLM01000000 (16309). The versions described in this paper are the first versions, BDLI01000000 (11226), BDLJ01000000 (11229), BDLK01000000 (11247), BDLL01000000 (15821), and BDLM01000000 (16309).
